# Assessing and Enhancing the Welfare of Animals with Equivocal and Reliable Cues

**DOI:** 10.3390/ani9090680

**Published:** 2019-09-13

**Authors:** Jason V. Watters, Bethany L. Krebs

**Affiliations:** San Francisco Zoological Society, San Francisco, CA 94132, USA

**Keywords:** anticipatory behavior, cognitive bias, judgment bias, human animal relationship, affective state, appetitive behavior, agency

## Abstract

**Simple Summary:**

Actions of human caretakers influence the experience of animals under their care, in zoos and elsewhere. These animals often learn to associate stimuli—sights, smells, sounds—with desirable outcomes such as feedings, training sessions, or other positive experiences. Here, we propose that a conscientious approach to providing reliable cues about daily events and observing animal behavior in response to both reliable and uncertain cues can help caretakers support and assess animal welfare.

**Abstract:**

The actions of human caretakers strongly influence animals living under human care. Here, we consider how intentional and unintentional signals provided by caretakers can inform our assessment of animals’ well-being as well as help to support it. Our aim is to assist in further developing techniques to learn animals’ affective state from their behavior and to provide simple suggestions for how animal caretakers’ behavior can support animal welfare. We suggest that anticipatory behavior towards expected rewards is related to decision-making behavior as viewed through the cognitive bias lens. By considering the predictions of the theories associated with anticipatory behavior and cognitive bias, we propose to use specific cues to probe the cumulative affective state of animals. Additionally, our commentary draws on the logic of reward sensitivity and judgement bias theories to develop a framework that suggests how reliable and equivocal signals may influence animals’ affective states. Application of this framework may be useful in supporting the welfare of animals in human care.

## 1. Introduction

There is an appreciable concentration on the development of animal welfare standards in much of the international zoo and aquarium community. Along with these initiatives is a call for measures of welfare and methods to ensure it. Zoo biologists are answering that call in many ways: the development of theory [1,2], multi-institutional studies of single species [3,4,5], case studies of individual animals [6,7], investigation of enrichment techniques [7,8,9,10,11], measurement of physiological responses [3,12,13,14], and more [15,16,17,18,19,20]. Often, the metric of animal well-being used is determined by the investigator’s definition of animal welfare.

For some welfare practitioners, animal welfare—like psychological well-being—is the individual’s own perception of their affective state [21,22,23,24,25,26,27]. A higher frequency of negative than positive experiences in one’s day to day life leads to feelings associated with a poor state of welfare while the converse state of emotional experiences supports feelings associated with positive welfare. This school of reasoning suggests that factors such as social environment, physical health and mental health moderate animal welfare inasmuch as they have an effect on the animal’s emotional state. In other words, the animal’s experience of these factors must result in negative affect for them to lead to poor welfare. An extension of the “affect as welfare” concept places animals themselves in control of managing their own emotional outcomes [22,28,29]. Increasingly, animal welfare scientists are finding support for the positive welfare implications of providing agency to animals under human care [22,23,30]. Such an approach is philosophically similar to the movement towards behavioral health in human health and social care [31]. In this arena, individuals are supported with opportunities to provide positive outcomes for themselves through education, intervention and facilitation of self-agency [32]—but how does one accomplish this with non-humans?

Humans very often drive the experiences of the animals in their care. Daily management activities by caretakers often provide animals with a variety of desirable outcomes (food, enrichment, access to new spaces), but can also be disruptive to the animal’s daily life [33]. Moreover, human attitudes towards the animals with which they work shape the manner of their interactions, and in turn the welfare outcomes for the animals [34,35,36]. Thus, because experience drives affect, outcomes associated with human-driven environmental changes and the human–animal relationship are likely to impact welfare. Researchers have studied the human–animal relationship and its effects on animal welfare for several decades [37]. The relationship occurs in any situation where animals rely on humans as a source of resources or as social partners. Investigations of the impact of the human–animal relationship occur in numerous settings including farms [35,38,39], homes [40,41] and zoos [34,42,43]. It is now widely accepted that how humans interact with animals—positively, negatively or ambivalently—impacts the emotional experience of animals and thus can drive welfare outcomes [39,40,41,42,43,44,45]. At the core of any relationship is communication and a basic element of communication is the signals or stimuli used to convey information [46,47]. Here we suggest that conscientious application of cues in all facets of animal husbandry will benefit animals and their caretakers in two ways. The first is that clarity of cues enhances animal welfare via supporting animals’ understanding of their environment and ability to make relevant decisions. The second benefit is that the behavioral responses of animals to both known and unknown cues provide human caretakers with insight into the animal’s perception of its state of well-being—that is, whether it perceives its experience as mostly positive or negative.

Here, we will use the term **cue** numerous times with various modifiers. Thus, we propose a few definitions of different types of cues. Because our focus is on human–animal interactions and their impact on animal behavior, we will reserve the use of the term cue for human generated signals. Specifically, we will use the term for signals that an animal might perceive that are generated by its direct caretaker. Conversely, we will rely on the term **stimulus** to refer to any other sensory signal present in the ambient environment. We realize that this is an arbitrary distinction of two words that are often considered synonymous, though we hope that it helps us to communicate clearly about information generated by animal caretakers and elsewhere.

**Figure 1:**
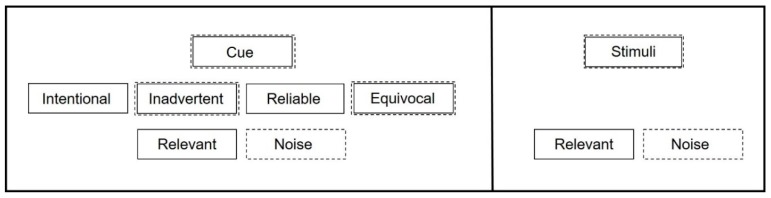
Different subsets of cues and their potential to provide relevance or become noise to animals. Solid boxes indicate types of cues with potential to provide relevance for animals. Dashed boxes indicate types of cues with potential to become noise. Ambient stimuli may also be relevant or noise to animals, but are not generated by human caretakers.

A **reliable cue** consistently precedes an event such that it indicates the coming of the event, and may be intentionally or inadvertently generated. The event may occur in the absence of the cue but the cue cannot consistently occur without preceding the event. Reliable cues prompt behavior associated with the event. This behavior may precede or follow the event. Reliable cues can be used by researchers or behaviorists to assess animal perception, determine animal preferences and observe anticipatory behavior. Announcements before a scheduled feeding or training session in a zoo setting would be examples of reliable cues [48,49]. An **equivocal cue** is a cue that is sensorily similar to two (or more) reliable cues that convey opposite information. For instance, an animal trained to recognize that a white shape precedes a reward and a black shape precedes a negative outcome might perceive a gray shape as equivocal (halfway between black and white). Equivocal cues can be used by researchers or animal welfare scientists to provoke behavioral responses aimed at assessing how the equivocal cue is perceived. The animal in the previous example would be expected to approach the gray shape if it perceived it as potentially positive, or to avoid it if the animal perceived it as possibly negative [49,50]. An **unattached equivocal cue** is a novel cue, and differs from an equivocal cue in that the animal has no learned associations with the unattached equivocal cue. The animal trained in the black/white shape paradigm could be presented with a novel audio cue or large red ball as unattached equivocal cues, as the animal would have limited expectation that such stimuli predict an event on the first presentation. Unattached equivocal cues can be used as response probes in several ways. One common use of the unattached equivocal cue is the novel object test. In a novel object test, animals are exposed to an object they have not previously encountered. Investigators record the animal’s response to the object, usually looking for evidence of approach or avoidance [51,52,53]. As the object is new, the animal has no learned associations with it, and the animal’s response can provide insight into its current affective state. Another behavioral response probe is the open field test, where there are many unattached equivocal cues and there can be multiple responses. For open field tests, animals are moved into an unfamiliar environment and placed in an exposed central area, away from shelter [54,55,56,57]. Researchers record whether the animal explores the open field or moves out of the unsheltered central area to the edge of the arena. Unattached equivocal cues are also used in learning paradigms such as training where a behavior is shaped with positive reinforcement. The unattached cue ultimately becomes a reliable cue that is attached to a specific outcome, such as when teaching an animal to associate a bridge with a primary reinforcer [58]. An **inadvertent cue** is one that was not purposefully given by the cue giver, who may be unaware of its existence, but which the animal perceives and has come to associate with a specific event [58,59]. An example of unintentional cues might be the sounds and smells of food preparation in areas adjacent to animal areas that drive anticipatory behavior long before mealtime. When we are referring equally to either a cue or a stimulus, we will use the term **signal**. **Noise**, as we will use the term here, is sensorily perceptible but conveys no actionable information for the animal. At its first perception, a noise may generate a behavioral response—as might an equivocal or unattached equivocal cue. Thus, both cues and stimuli have the potential to become noise or provide relevant information to animals (Figure 1).

One might imagine that any individual’s day is filled with a combination of reliable, equivocal, unattached equivocal cues, unintentional cues, stimuli and noise. A **signal string** is the admixture of cues, stimuli and noise—in no particular order—that may precede any event. The frequencies of these different types of sensory information that an individual perceives likely drives its perception of its understanding of the world and its ability to manage its behavior in response to this relevant information, thus influencing its welfare. From the individual’s perspective, there is a drive to convert equivocal and unattached equivocal signals to reliable ones and to understand what is a signal and what is noise.

## 2. How Do Cues Relate to Animal Welfare?

Cues and stimuli are closely tied to an animal’s welfare because they help the animal to understand the nature of a coming experience—whether the experience will positively or negatively alter its affective state. This ability to act with self-agency is considered to support animal welfare and has been suggested to be a highly motivated behavioral need with support from various studies ranging from contra-freeloading to cognitive task solving [10,28,60,61,62,63]. The positive welfare impacts of self-agency have been most extensively studied in mammals and birds, however it is worth noting the apparent lack of evidence supporting its importance in other taxa may be largely due to limited research into reptile, amphibian or invertebrate welfare overall [64,65]. Understanding what is coming and being able to respond also allows one to avoid aversive events and take advantage of positive ones.

In an interesting study, Rimpley and Buchanan-Smith demonstrated that reliably cueing an event that was apparently negative to a group of capuchin monkeys—cage cleaning—reduced the negative impact of this practice on the animals [66]. Prior to implementing the reliable cue, the authors determined that the frequency of anxious behaviors was relatively high when the monkeys could not determine exactly when cage cleaning would occur. They interpreted this to suggest that the monkeys found cage cleaning to be aversive. After application of a reliable cue that indicated to the monkeys when cage cleaning would occur, the frequencies of these behaviors decreased.

In a review of the effects of predictability and unpredictability of aversive and positive events, Bassett and Buchanan-Smith show that animals tend to choose predictability over unpredictability of events [66]. They also state that the data suggest that temporally unpredictable but reliably cued (they use the term signaled) aversive events appear to be more preferable to animals than temporally predictable aversive events. Perhaps this is because animals can develop a sense of when to expect temporally predictable events and cannot develop this sense when these events can occur at varying times. These authors also show that animals also prefer temporally unpredictable but reliably cued appetitive events over un-cued but temporally predictable appetitive events. They note that temporal unpredictability of appetitive events may enhance animal welfare as it may reduce boredom. Thus, it appears that a combination of predictability and unpredictability may be effective at satisfying motivation associated with appetitive behavior. Bassett and Buchanan-Smith’s primary conclusions are that a loss of predictability negatively affects welfare and that clarity of cues supports animal welfare [66].

## 3. How Do Cues Help Us Understand Welfare?

The list of behaviors that describe animals’ responses to positively and negatively valenced events is growing. Research indicates that many types of behavior from play to self-grooming may relate to momentary emotional states [21,27,67,68,69]. Welfare behaviorists have made progress in interpreting animals’ actions and how these reflect animals’ perceptions of their own affective state. For example, several studies of non-human primates and humans as well indicate that self-directed behaviors such as autogrooming are related to feelings of anxiety [70]. Yet, there remains some question as to whether specific behaviors are useful indicators of cumulative affective state. Two specific types of behavior that are currently under study as potential indicators of more cumulative states of affect are anticipatory behavior and decision-making behavior.

Several researchers consider anticipatory behavior to be an indicator of reward sensitivity or a reflection of the cumulative balance between positive and negative experiences [2,48,71,72,73]. It is a taxonomically widespread phenomenon and tends to present as increased activity and attentiveness occurring in the time between the detection of a signal that is associated with the likely acquisition of a reward and the consummatory act [7,71,74,75,76]. Numerous studies have demonstrated that anticipatory behavior can be modulated [7,73,77,78]. Krebs and Watters added a cognitive enrichment to an animal’s enrichment program and noted a decrease in the intensity of anticipatory behavior [7]. Although anticipatory behavior can be stimulated by internal mechanisms such as a learned schedule, it is easiest to apply external cues to clearly delineate timing of the behavior for study and potential welfare assessment [6]. Introduction of a reliable cue that indicates a coming positive event provides a means to elicit the behavior and describe it. The technique can be used to determine if the animal views the coming event as positive (as in [6]) and also to assess the intensity of anticipatory behavior—measured by speed, transition rate, or other similar measures. Knowledge of what animals perceive to be rewarding helps to develop strategies for how to enrich animals’ lives. The theory describing how anticipatory behavior indicates reward sensitivity suggests that the intensity of the behavior increases with the frequency of negative events in an animal’s experience. This increased intensity of behavior is assumed to reflect the animal’s reward sensitivity which increases as welfare decreases—though with extremely poor welfare, the animal is predicted to stop expressing anticipatory behavior and become anhedonic [2,79].

We suggest that anticipatory behavior is a regular part of an animal’s day as it is appetitive behavior and much of an animal’s activity is aimed at acquiring rewards. Studies indicate that the individual behaviors that comprise anticipatory behavior are numerous and that animals transition between these behaviors, sometimes frequently, while expressing anticipatory behavior [2,6,7,43,48,72,80,81]. They are essentially behaviors that are used to track and follow signals in support of acquisition of a sought reward. Because the number of behaviors performed in a single bout of anticipatory behavior and the intensity of those behaviors may vary, we suggest using the term concentration to refer to the combination of the number of individual behaviors and intensity of those behaviors in a bout of anticipatory behavior.

**Figure 2:**
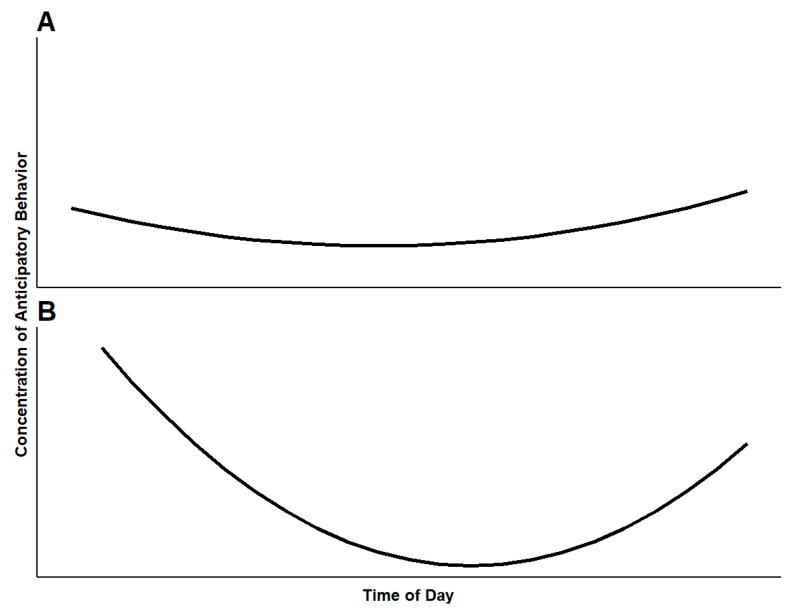
Concentration of anticipatory behavior expressed by animals living in (**A**) a high quality environment with numerous positive opportunities available throughout the day and (**B**) a low quality environment with positive opportunities only early and late in the day. This model assumes caretakers provide routine husbandry (feed and enrichment) at least twice early and late in the day, but in (**A**) caretakers provide more opportunities in addition to routine servicing spread throughout the day.

For animals in high quality environments with numerous positive opportunities, we expect the maximum concentration of anticipatory behavior to be lower overall than that of animals in lower quality environments (Figure 2A). This relates to the animal’s state of reward sensitivity. Animals living in high quality environments likely have many positive behavioral opportunities spread throughout the day, and thus have many chances to express anticipation of positive events. Such animals will have lower reward sensitivity than an animal with fewer opportunities, and are likely to exhibit less intense anticipation towards any single positive event. Due to the availability of positive events throughout the day, the animal may exhibit low-intensity anticipation throughout the day [2]. For animals in high quality environments, anticipatory behavior may be difficult to observe as it is likely to be a part of a diverse time budget. In low quality environments, animals should show highly concentrated anticipatory behavior at infrequent times (Figure 2B). In many cases, animals under human care will receive food, enrichment, or changes to their housing once or twice a day. Figure 2 assumes a twice daily servicing routine, with positive opportunities occurring at least twice, early and late in the day. Anticipatory behavior decreases in intensity upon receiving the desired reward. As such, the curves in Figure 2A,B are concave in appearance, as the concentration of anticipatory behavior towards a coming reward will drop off once the reward arrives. This effect would be the most pronounced for animals in low quality environments, which would have few positive events to anticipate in between caretaker visits (Figure 2B). This would decrease the concentration of anticipatory behaviors to much lower levels than for animals in higher quality environments (Figure 2A). Here, the use of a reliable cue aids in describing anticipatory behavior. For assessing welfare using anticipatory behavior, the concentration of the behavior of two or more animals in the same conditions can be compared or an attempt can be made to modulate the behavior of a single animal so that the baseline expression of the behavior can be compared to a new concentration. Changes in concentration can be assessed in response to various management changes. Comparisons of this kind may be most effectively applied to changes with a clear expectation of positive or negative outcomes for animals. For example, assessment of concentration before and after disruption of a social group, or before and after addition of new enrichment protocols.

Another class of behavior that appears to reflect a more cumulative affective state is decision-making behavior. Similar to the way in which anticipatory behavior appears to demonstrate whether underlying motivations or needs have been met, decision-making behavior in response to equivocal signals reveals optimistic or pessimistic states of mind [15,17,80,82,83]. Animals that have experienced positive opportunities, such as those living in highly enriched environments express apparently optimistic behavior upon investigating an equivocal cue while those from poor environments are less likely to investigate when offered such a cue and appear pessimistic towards it. This seeming cognitive bias towards optimism or pessimism appears to be driven by long-term emotional state and is assessed with a judgment bias test.

In a judgment bias test, animals are given reliable cues for a reward and an aversive or unrewarding stimulus [15,17,82,84]. The cues are often similar but clearly discernible. For example, they may be a black and white cue, two different tones or opposite locations in a room. The animal learns each cue is 100% reliable for the outcome it signals. The positive cue will always be followed by a reward, and the negative cue will either be followed by no reward or an unpleasant event. The probability of outcomes is certain for the known cues. A trained animal is then presented with an equivocal cue—typically one that is similar to both reliable cues—perhaps a grey cue, a tone that is a frequency between the two reliable tones or a position between the two reliable locations. The animal has no prior knowledge of what outcome this cue represents, nor any expectation of the cue’s reliability. An animal approaching an equivocal cue is therefore expressing an expectation of a positive outcome and is said to be acting optimistically.

Judgment bias studies will often adjust animals’ welfare state in either a positive or a negative direction, typically by placing some animals in a high quality environment and others in a poor quality environment [85,86]. After this adjustment, animals whose welfare has been adjusted positively act optimistically towards equivocal cues while those who have received a negative adjustment act pessimistically. Cognitive bias tests are useful for assessing animal’s perception of their cumulative affective state because the cues used and reward probabilities are tightly controlled. Similar to using anticipatory behavior to assess the cumulative affective state’s effect on reward sensitivity, cognitive bias assumes that the underlying emotional state of an animal drives its expression of decision-making behavior. Cognitive bias tests allow investigators to vary the outcome while maintaining cue reliability. The animal has reason to expect an equivocal cue indicates an event, however it does not know whether the event will be positive or negative.

**Figure 3:**
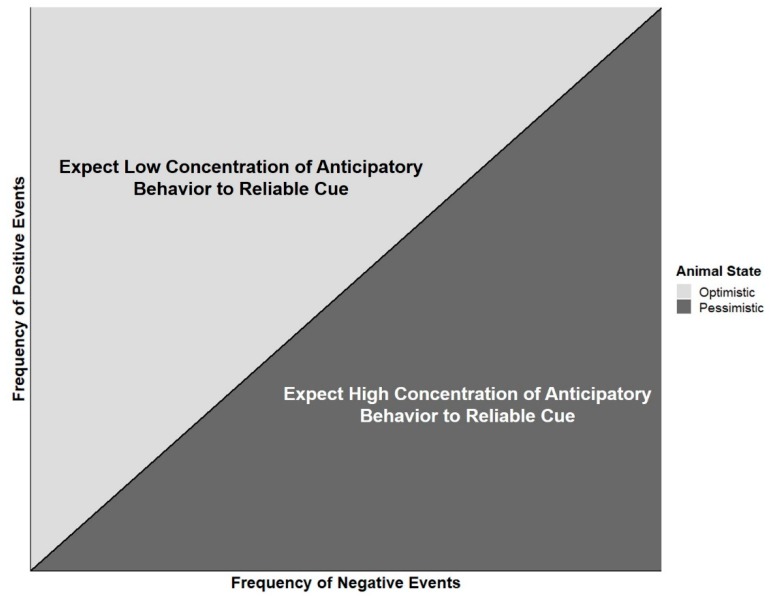
Graphical model of how the ratio between the frequency of positive and negative events influences animal responses to reliable (anticipatory response) or equivocal (optimistic or pessimistic judgment bias response) cues.

We suggest that anticipatory behaviors and optimistic behaviors are related appetitive behaviors. While anticipatory behavior is described in relation to a reliable cue and optimistic cognitive bias is assessed with an equivocal cue, the two are clearly aimed at acquisition of a reward. One study has demonstrated that dolphins who express more anticipatory behavior prior to a positive event also respond in a more pessimistic fashion in a cognitive bias test [80]. We further propose that cue reliability and equivocality are key to determining animals’ underlying cumulative emotional state and that using a combination of cues as probes to assess welfare has potential for furthering our understanding of these behaviors. Figure 3 shows a simple graphical model that describes behavioral predictions of animals presented with either a reliable or an equivocal cue. For simplicity, consider that this model reflects the range of welfare states described in the predictive model of anticipatory behavior that Watters [2] modified from van der Harst and Spruijt [79] up to but not including the range where chronic stress occurs. With a cumulative high proportion of positive to negative events, animals are expected to express a relatively low concentration of anticipatory behavior towards reliable cues, but also to act optimistically towards equivocal ones. Conversely, when animals experience an events ratio that skews negatively, they are expected to ignore equivocal cues and pay close attention to those which their experience indicates lead to positive outcomes.

**Figure 4:**
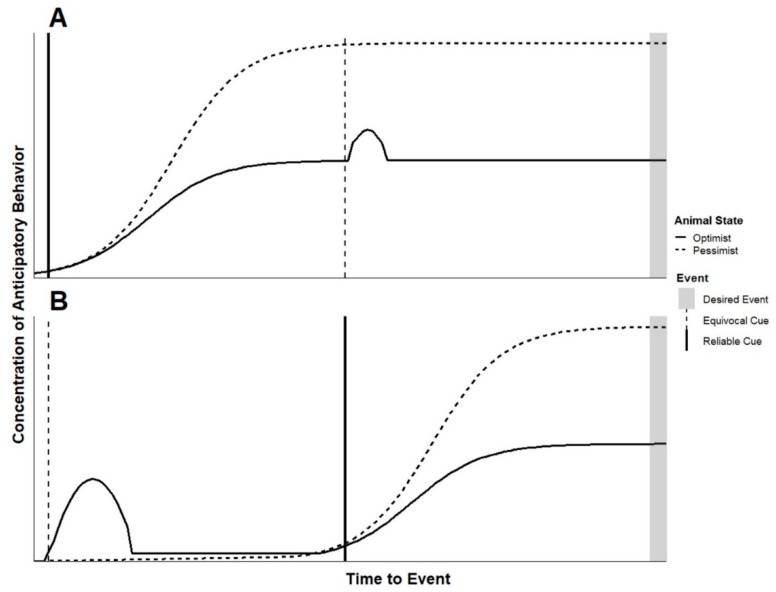
Predicted responses of animals in an optimistic or pessimistic state to (**A**) a reliable cue for an event followed by an equivocal cue and (**B**) an equivocal cue followed by a reliable one.

While precise application of specific sorts of cues promises to lead to a deeper understanding of animal welfare, in practice, those looking for quick assessments should consider that nearly all signals occur within a signal string. Animals’ responses to cues used to probe their welfare state should be considered within this context. The composition of signal strings of similar duration can vary in terms of the frequency of and types of signals in the string. For example, a simple signal string may include a reliable cue followed by the expected event (e.g., animal hears a door open, caretaker presents food) while a more complex string could include several other types of signals between the cue and the event (e.g., animal hears a door open, caretaker prepares food in nearby space, goes in and out several times, services another animal and then presents food). Figure 4 provides some insight into interpreting observations in these non-experimental contexts.

For this model, it is important to consider potential behavioral differences between animals in a pessimistic state resulting from repeated aversive experiences compared to animals in a pessimistic state resulting from infrequent positive experiences. The management of the animal in question will likely dictate which of these situations it experiences, and this has direct implications for judgment biases towards equivocal cues. Both applying aversive stimuli and withholding reward have been successfully used to assess cognitive bias in animals [15]. In some cases, repeated aversive stimuli are applied to animals in periods outside of cognitive bias testing specifically to induce anhedonia in the animal (e.g., mild chronic stressor [87,88]), however this approach is largely not used for obvious reasons when assessing welfare. Laboratory animals experiencing daily invasive procedures compared to the same species only disturbed for daily feedings may both respond pessimistically on a cognitive bias test by not approaching an equivocal cue, however animals conditioned to expect aversive outcomes may exhibit heightened vigilance towards novelty [89,90]. In discussion of the simplified signal string presented in Figure 4, we are assuming the pessimistic animal in question has limited positive opportunities throughout its day and has not been subjected to repeated aversive events. Under this assumption, we expect an optimistic animal may more readily respond behaviorally towards multiple equivocal signals in a signal string—even while still anticipating a known positive event—while a pessimist may perceive the same signals yet remain focused on anticipating the known reward.

Figure 4 provides predictions regarding the anticipatory behavior response expected when animals of either mostly positive or mostly negative experience encounter a simple signal string. Here, in addition to a reliable cue for a positive event, the signal string includes an equivocal cue—one which will ultimately become noise as it is associated with no event but is used as a probe. In these simple signal strings, the equivocal cue occurs either before or after the reliable cue and the onset of anticipatory behavior. It is important to note the equivocal cue should be similar in sensory modality to signals the animal is known to respond to, thus ensuring the animal perceives it within a known paradigm of cues. In both cases, we expect the equivocal cue to prompt appetitive behavior in the animal with mostly positive experience. In the case where the equivocal cue follows the reliable cue, this may appear as a brief uptick in anticipatory behavior, as the optimistic animal would be expected to respond as if the equivocal cue indicates an additional opportunity for reward. We also expect the animal with mostly negative experiences to perceive and avoid an equivocal cue, continuing to express a relatively high concentration of anticipatory behavior in response to the known positive cue. In practice, we imagine that equivocal cues can be used as probes to further understand the welfare state of animals that are already expressing anticipatory behavior or outside of the times when anticipatory behavior is expected.

## 4. How Do Cues Support Welfare?

**Figure 5:**
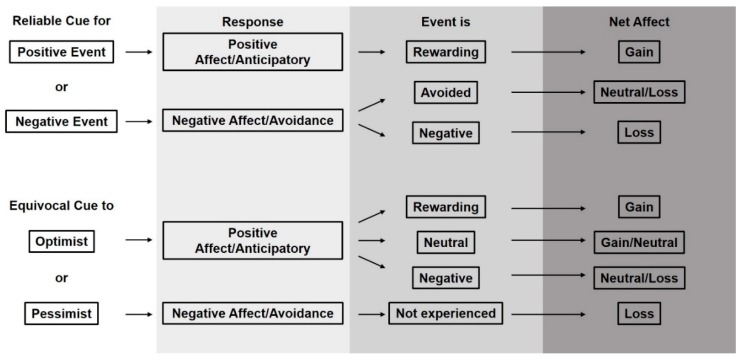
Predicted net welfare impacts of animal responses to reliable cues and equivocal cues for different types of events (rewarding, neutral, or negative).

We suggest that animals will benefit if their caretakers attempt to clearly communicate to them when important events will happen, be they positive or negative. Similarly, we contend that a high frequency of equivocal cues can negatively affect animals’ psychological state. Here, we frame an argument describing how cues and their associated outcomes can drive psychological states of animals (Figure 5). Building from our understanding of how animals with varied cumulative psychological states (e.g., optimistic vs pessimistic) perceive reliable and equivocal cues, our framework describes predictions for how cues and their associated events modify the cumulative emotional state of an individual one experience at a time.

Firstly, for all cumulative states of affect, except animals experiencing chronic stress and related anhedonia, reliable cues for either positive or negative events should elicit predictable behavioral and psychological results. A reliable cue indicating a coming positive event results in the animal expressing anticipatory behavior and an associated positive emotional response that is further supported upon consummation [71,81,91] (Figure 5; Reliable Cue for Positive Event). Similarly, but on the opposite end of the emotional spectrum, a reliable cue for a negative event should elicit a negative emotional response which leads to avoidance behavior, or provides animal context for when to expect an aversive event if it is unavoidable. It is possible that if this avoidance is successful, the animal gains some positive feeling for having successfully dealt with a stressor [60] (Figure 5; Reliable Cue for Negative Event). In the instance that the animal is unable to avoid the reliably cued negative event, it likely experiences further negative emotion [66].

Secondly, considering animals with alternative cumulative states of affect, cognitive bias research indicates that those with mostly positive experience will respond differently to an equivocal cue than those with mostly negative experience. This difference in response is similar to the way in which animals react to reliable cues. An animal with mostly positive experience approaches the equivocal stimulus in a fashion similar to the animal who expresses anticipatory behavior following a reliable cue for a positive event. Here, investigation is a type of appetitive behavior and like anticipatory behavior, is rewarding in itself [92]. If the questionable cue in turn leads to a positive event, then the animal experiences a similar reinforcing emotional response as the one who expresses anticipatory behavior upon noting a reliable cue for a positive event and then achieves consummation of that event (Figure 5; Equivocal Cue to Optimist). For equivocal cues that do not lead to positive events, the emotional outcomes differ. For the same animal as above with mostly positive experiences, investigating the equivocal cue may indeed be rewarding but if that leads to a negative event, then the animal is faced with a negative emotional experience that may negate the prior positive moment. In the end, and if the negative experience is strong enough, the animal may be worse off emotionally than prior to responding to the equivocal cue (Figure 5; Equivocal Cue to Optimist). Another alternative for this animal is that the equivocal cue lead to no event and is in effect noise. In this case, the emotional outcome may be neutral as investigation leads to no reward or perhaps slightly positive as knowledge gain may be rewarding [10] though this slight reward may not be repeatable. For the animal with mostly negative experience, cognitive bias theory suggests that an equivocal cue may be perceived as an indicator of a negative outcome and thus generate negative emotion and be avoided (Figure 5; Equivocal Cue to Pessimist).

Given this framework, it appears that uncertainty of information content of signals can lead animals to experience negative affect in two primary ways. The first of these ways occurs at perception of the signal. If the animal perceives the signal to be related to negative events, then the animal experiences a negative emotional outcome associated with perceiving the signal. The second way equivocal signals may influence negative affective outcomes is that they may lead to negative experiences. Thus, caretakers may strive to develop information rich environments and husbandry plans for animals. Such plans would aim to reduce the ratio of equivocal and unattached equivocal cues to reliable cues to support animals having more useful than useless information about their environment and to facilitate an increased frequency of positive experiences for animals who may currently be in a pessimistic state. Pessimists may, overall, experience greater welfare benefits from the addition of any reliable cue to their environments, as additional information supports informed decision making and allows risk averse individuals more agency over their experiences.

Related to the addition of reliable information to animals’ signal strings, it is worth considering that many of the signals that animals under human care perceive are related to humans’ schedules. It is possible that these inadvertent strings become somewhat or mostly reliable from the animals’ perspective. Unintentionally reducing information available to animals who may have come to rely on these strings for information—through abrupt schedule changes, habitat modifications or the addition of sensory noise that prevents the perception of signals that animals rely upon, may have temporary negative affective outcomes for animals. If these changes are recurring, following a longer schedule than animals’ regular daily schedule, for example weekly or even less frequently, they could be viewed by caretakers as regular and potentially avoidable stressors.

## 5. Discussion

All relationships benefit from clear communication and the one between an animal living in a zoo and its caretakers is no different. Here, we have furthered the argument in favor of providing animals whose lives include a significant relationship with humans with clear understanding of when important human-driven events will happen. In doing so, we have extended theory regarding anticipatory behavior and judgment bias to demonstrate their relatedness and how animals’ responses to various types of cues can help to elucidate their cumulative emotional state.

Various signals can be used to provoke animals’ behavioral responses to them—and with an understanding of how animals in different emotional states respond to different types of signals, cues can be used as probes to gain insight into the emotional well-being of animals. While we have not focused on providing a “how-to guide” for employing cues to assess and promote animals’ behavioral health, we have provided definitions of various types of signals and predictions for how they might prompt behavioral responses that reflect the cumulative affective state of the animal of interest.

The relationship between animal caretakers and animals extends beyond direct interactions. Animal caretakers may benefit from achieving a complete understanding of how their routines are “listened to” by animals, how other factors affect animals’ ability to predict those routines and the outcomes associated with them. Outside of the routine, animals may also benefit from a notification of when to expect events that may otherwise come as a surprise. Such notification may prompt investigatory behavior for positive events like a foraging opportunity or preparatory behavior for negative events such as restraint for medical procedures.

We have provided a framework for how signals and their associated events may affect the psychological state of animals one experience at a time. We suggest that in some cases, these outcomes vary for animals in different states of mind and that in many cases, unclear information itself leads to negative welfare outcomes. There are numerous cases where animals may rely on inadvertent cues as sources of information relevant to them. It is possible that these inadvertent cues are unintentionally modified or masked in the course of daily work by caretakers (e.g., inadvertent audio cues animals rely on are masked by ventilation system noise). Consideration of this possibility may help animal caretakers to understand why or when an animal’s behavior appears to suddenly change.

We appreciate that when theory is extended, so too are the questions. For example, how does one apply an equivocal cue in the midst of an ongoing signal string. Perhaps the answer there lies in assessing the types of signals present and looking for a novel but similar cue. Considering animals’ sensory capabilities will likely be of use. Another question: we have discussed behaviors that are presumed to reflect animals’ cumulative emotional state, but what exactly is the time-frame for “cumulative?” We hope that the ideas developed here stimulate research as well as help to support applied animal behaviorists in their clinical assessment of animals.
